# The effect of early, comprehensive genomic testing on clinical care in neonatal diabetes: an international cohort study

**DOI:** 10.1016/S0140-6736(15)60098-8

**Published:** 2015-09-05

**Authors:** Elisa De Franco, Sarah E Flanagan, Jayne AL Houghton, Hana Lango Allen, Deborah JG Mackay, I Karen Temple, Sian Ellard, Andrew T Hattersley

**Affiliations:** a Institute of Biomedical and Clinical Science, University of Exeter Medical School, Exeter, UK; b Wessex Regional Genetics Laboratory, Salisbury Foundation Trust, Salisbury, UK; c University Hospital Southampton NHS Trust, Southampton, UK; d Human Genetics and Genomic Medicine, Faculty of Medicine, University of Southampton, Southampton, UK

## Abstract

**Background:**

Traditional genetic testing focusses on analysis of one or a few genes according to clinical features; this approach is changing as improved sequencing methods enable simultaneous analysis of several genes. Neonatal diabetes is the presenting feature of many discrete clinical phenotypes defined by different genetic causes. Genetic subtype defines treatment, with improved glycaemic control on sulfonylurea treatment for most patients with potassium channel mutations. We investigated the effect of early, comprehensive testing of all known genetic causes of neonatal diabetes.

**Methods:**

In this large, international, cohort study, we studied patients with neonatal diabetes diagnosed with diabetes before 6 months of age who were referred from 79 countries. We identified mutations by comprehensive genetic testing including Sanger sequencing, 6q24 methylation analysis, and targeted next-generation sequencing of all known neonatal diabetes genes.

**Findings:**

Between January, 2000, and August, 2013, genetic testing was done in 1020 patients (571 boys, 449 girls). Mutations in the potassium channel genes were the most common cause (n=390) of neonatal diabetes, but were identified less frequently in consanguineous families (12% in consanguineous families *vs* 46% in non-consanguineous families; p<0·0001). Median duration of diabetes at the time of genetic testing decreased from more than 4 years before 2005 to less than 3 months after 2012. Earlier referral for genetic testing affected the clinical phenotype. In patients with genetically diagnosed Wolcott-Rallison syndrome, 23 (88%) of 26 patients tested within 3 months from diagnosis had isolated diabetes, compared with three (17%) of 18 patients referred later (>4 years; p<0·0001), in whom skeletal and liver involvement was common. Similarly, for patients with genetically diagnosed transient neonatal diabetes, the diabetes had remitted in only ten (10%) of 101 patients tested early (<3 months) compared with 60 (100%) of the 60 later referrals (p<0·0001).

**Interpretation:**

Patients are now referred for genetic testing closer to their presentation with neonatal diabetes. Comprehensive testing of all causes identified causal mutations in more than 80% of cases. The genetic result predicts the best diabetes treatment and development of related features. This model represents a new framework for clinical care with genetic diagnosis preceding development of clinical features and guiding clinical management.

**Funding:**

Wellcome Trust and Diabetes UK.

## Introduction

**Figure 1:**
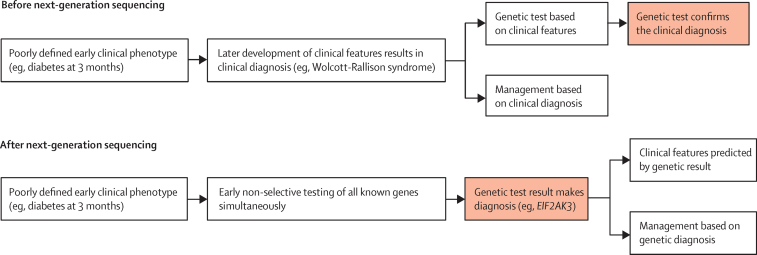
The paradigm shift of genetic testing Schematic representation of the steps involved in genetic testing before and after the introduction of next-generation sequencing. The red boxes indicate the role of genetic testing.

The traditional genetic testing approach is based on the selection of a gene or genes that are tested according to clinical phenotype. This approach places an emphasis on the recognition of clinical syndromes and results in mainly confirmatory genetic testing. The advent of next-generation sequencing has the potential to revolutionise the approach to diagnostic genetic testing, because both exome sequencing and custom gene-panel tests for specific disorders analyse all genes known to cause a genetically heterogeneous disorder in one test (figure 1).

Neonatal diabetes diagnosed before 6 months of age is a rare (incidence about one in 100?000 livebirths),^1^, ^2^ genetically heterogeneous disease, typically caused by mutations in one gene. By contrast, diabetes diagnosed after 6 months is most likely to be type 1 diabetes with an autoimmune cause.^3^, ^4^ 22 known genetic causes of neonatal diabetes exist (mutations in 21 genes and methylation abnormalities at the 6q24 locus) that identify different clinical subtypes of the disease. This includes isolated permanent neonatal diabetes, transient neonatal diabetes, and complex syndromes in which neonatal diabetes is often the presenting feature (eg, Wolcott-Rallison syndrome). Traditional genetic testing for neonatal diabetes requires accurate clinical information about the patient's phenotype to allow selection of a small number of genes to test. Because neonatal diabetes is generally the presenting feature of the disease, this approach is restricted by the clinical information available at the time of referral and relies on timely clinical updates on the subsequent development of additional features from the referring clinicians.

The most common causes of neonatal diabetes are mutations in the potassium channel subunit genes *ABCC8* and *KCNJ11.*^5^, ^6^, ^7^ Patients with neonatal diabetes caused by a potassium channel gene mutation are sensitive to sulfonylurea treatment and therefore their clinical management can be improved by replacing insulin with oral agents.^8^, ^9^, ^10^, ^11^ This situation highlights the importance of early genetic diagnosis in neonatal diabetes, and international guidelines suggest immediate referral for genetic testing as soon as a clinical diagnosis of neonatal diabetes is made.^12^

Three targeted next-generation sequencing assays have been developed for genetic testing of monogenic diabetes,^13^, ^14^, ^15^ including our panel that includes all the known neonatal diabetes genes.^14^ A methylation assay is needed to detect 6q24 abnormalities. We used the targeted next-generation sequencing assay to test all the genetically undiagnosed patients in a large, international cohort of 1020 patients with diabetes with the objective of assessing the effect of early, comprehensive genetic testing in this disease.

Panel **Research in context**

**Systematic review**

Between Jan 1, 2000, and Jan 1, 2014, we searched PubMed with the MeSH search terms “Neonatal Diabetes” OR “PNDM” OR “TNDM” AND “genetic testing” OR “sequencing” OR “mutation” for articles published before Jan 1, 2014, describing genetic testing in neonatal diabetes. None of the articles identified by this search assessed the results and effect of comprehensive genetic testing in patients with neonatal diabetes. We found two studies describing the development of targeted next generation sequencing approaches for genetic testing in neonatal diabetes,^13^, ^15^ but they only described their use in small validation studies. The large study of 174 neonatal diabetes patients by Busiah and colleagues^32^ reported the analysis of the four commonest of the known genetic causes. Several studies have described the improvement in glycaemic control in patients with a *KCNJ11* or *ABCC8* mutation after transferral from insulin to sulfonylurea therapy.^7^, ^8^, ^9^, ^10^ We found no data to suggest whether the progress in genetic testing has resulted in reduced time from diagnosis of diabetes to referral for genetic analysis during the past decade or the effect of early genetic diagnosis on the clinical management of patients. Therefore, we undertook comprehensive testing in a large cohort of patients with neonatal diabetes to assess how an early genetic diagnosis might affect clinical care.

**Interpretation**

Our findings provide an accurate estimation of the molecular contribution and clinical features of the known genetic causes of neonatal diabetes. Patients are referred for genetic testing soon after diagnosis, often when they only have isolated diabetes. Our results show that early comprehensive genetic testing allows identification of the underlying genetic defect in about 82% of patients. The genetic diagnosis will inform clinicians on the probable course and best management of the patient's diabetes and the likely future development of additional clinical features. This represents a new framework for clinical care in neonatal diabetes, with genetic diagnosis often preceding development of clinical features and guiding clinical management.

## Methods

### Patients

Between January, 2000, and August, 2013, genetic testing was done in 1020 patients (571 boys, 449 girls) diagnosed with diabetes before 6 months of age who were referred to the Exeter Molecular Genetics laboratory from 79 countries. We excluded 85 patients for whom there was insufficient DNA available for comprehensive testing (appendix). Clinical information was provided by the referring clinicians from clinical notes via completion of a neonatal diabetes request form. Genetic testing for neonatal diabetes was offered free of charge. The study was done in accordance with the Declaration of Helsinki principles with informed parental consent given on behalf of children.

The median age at diagnosis of diabetes was 6 weeks (IQR Q1–Q3 1–12), and the median birthweight was 2460 g (median standard deviation score [SDS] –1·7 [–2·6 to 0·8], median centile 4·7 [0·5–22·8]). We defined patients born to consanguineous parents as those whose parents were first or second cousins (n=215), or those from countries with a high prevalence (>20%) of consanguineous unions^16^ (appendix) in whom genome-wide SNP typing showed genomic homozygosity of 1·56% or higher,^17^ as previously described^18^ (n=15; appendix). We used the Chi-square test to assess the differences between the consanguineous and non-consanguineous groups.

### Molecular genetic testing

All patients were tested until a causative mutation was identified by Sanger sequencing or targeted next-generation sequencing^14^ for all 21 known neonatal diabetes genes or by methylation analysis for chromosome 6q24 abnormalities. The appendix shows the current testing pipeline.

Initial analysis consisted of rapid (<2 weeks) Sanger sequencing of *KCNJ11, ABCC8*, and *INS*^19^ with testing for chromosome 6q24 abnormalities by methylation analysis^20^ in patients with a clinical diagnosis of transient neonatal diabetes, or those patients younger than 6 months at the time of testing. Since 2012, this initial testing was followed by comprehensive testing of all other genes by the targeted next-generation sequencing assay. Before 2012, additional genes were sequenced by Sanger sequencing according to clinical features or as part of a cohort analysis when a new gene was discovered and the phenotype needed to be defined.

In all patients for whom a genetic diagnosis had not been identified by this initial testing, we used a custom targeted next-generation sequencing panel^14^ to sequence all known neonatal diabetes genes (appendix). This assay used the Agilent SureSelect in solution capture system. We adjusted bait density and replication from Agilent (version 1) exome capture data to achieve more even coverage over the targeted genes. A minimum coverage of 30 reads per base for the coding region ±50 bp was achieved for 98·5% of bases. Partial or whole gene deletions or duplications were identified by relative read depth coverage as previously described.^14^ The mutations identified by targeted next-generation sequencing were confirmed by Sanger sequencing or multiplex ligation-dependent probe amplification.

We used the bioinformatic method ALAMUT (Interactive Biosoftware, Rouen, France) to predict the effect of novel variants. We used microsatellite analysis of parent and proband trios using the PowerPlex 16 kit (Promega, Southampton, UK) to confirm biological associations in probands with apparently de-novo mutations. 253 (25%) of the 1020 patients had been included in previous publications (appendix).

### Role of the funding source

The funders of the study had no role in the study design, data collection, data analysis, data interpretation, or writing of the manuscript. The corresponding author had full access to all the data in the study and had final responsibility for the decision to submit for publication.

## Results

**Figure 2:**
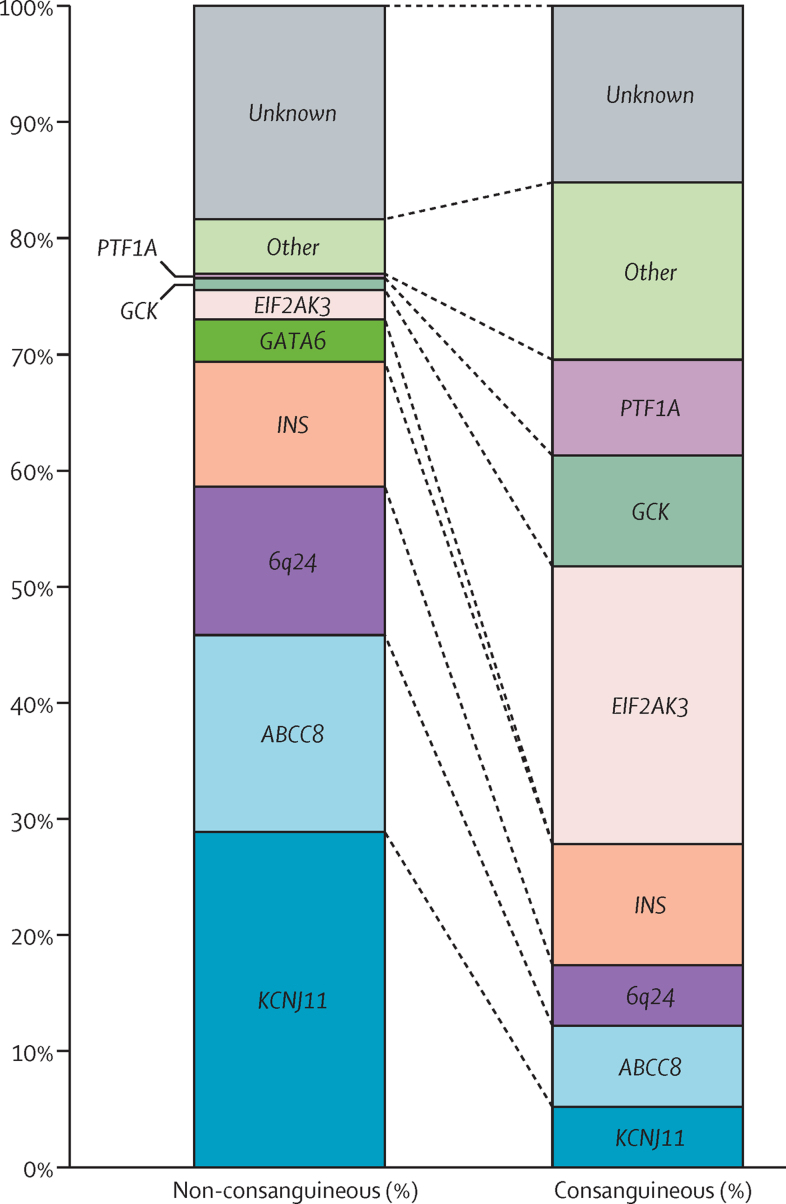
Different genetic causes of neonatal diabetes in patients born to non-consanguineous and consanguineous parents Comparison of genetic causes of neonatal diabetes in non-consanguineous (n=790) and consanguineous groups (n=230). Consanguinity is defined by parents being second cousins or more closely related or by the presence of 1·56% or higher total homozygosity.^17^ Genes mutated in fewer than 2·5% of patients in both cohorts were grouped in the other category (appendix).

We identified the genetic cause of neonatal diabetes in 840 (82%) of 1020 patients. The proportion of patients for whom a genetic diagnosis could not be identified was similar in patients born to unrelated parents and related parents (18% in infants with non-consanguineous parents *vs* 15% with consanguineous parents; p=0·27; df=1). These data suggest that both dominant and recessive causes of neonatal diabetes are still undiscovered (figure 2 and appendix).

Parental consanguinity made a striking difference to the mode of inheritance of neonatal diabetes (figure 2). The major difference was that, as expected, recessive causes were common (158 [81%] of 195 patients) in the offspring of consanguineous parents but unusual (81 [13%] of 645 patients) when the parents were not consanguineous (p<0·0001; df=1). Dominant heterozygous mutations were more common (457 [71%] of 645 patients) in the offspring of non-consanguineous parents and most of them were spontaneous (216 [68%] of 320 patients when both parents were available for testing).

The major genetic cause differed depending on whether the parents were related. Mutations in *KCNJ11*^5^ and *ABCC8*^6^, ^7^ accounted for 46% of ca*s*es in the non-consanguineous cohort, but only 12% in the consanguineous group (p<0·0001; df=1). This is an important diagnosis because glycaemic control can be substantially improved for most of these patients by transferring from insulin injections to high-dose sulfonylurea tablets.^9^, ^11^ In patients born to consanguineous parents, the most common cause (56/230 [24%]) of neonatal diabetes was a homozygous mutation in the *EIF2AK3* gene causing Wolcott-Rallison syndrome.^21^

Mutations in the *INS* gene were present at a similar proportion in the two groups (11% of patients with non-consanguineous parents *vs* 10% of infants with consanguineous parents; p=0·89; df=1), but the mechanism underlying the disease is fundamentally different. In the non-consanguineous group, most *INS* gene mutations were heterozygous (77 [90%] of 86 patients) and affected the structure of the preproinsulin protein.^22^ Data from mouse models and in-vitro studies suggest that these mutations result in altered protein folding causing severe endoplasmic reticulum stress and ultimately ß-cell destruction. Of patients born to consanguineous parents, *INS* mutations were mainly (18 [75%] of 24 patients) homozygous loss-of-function changes that impaired the synthesis of insulin.^23^

**Figure 3:**
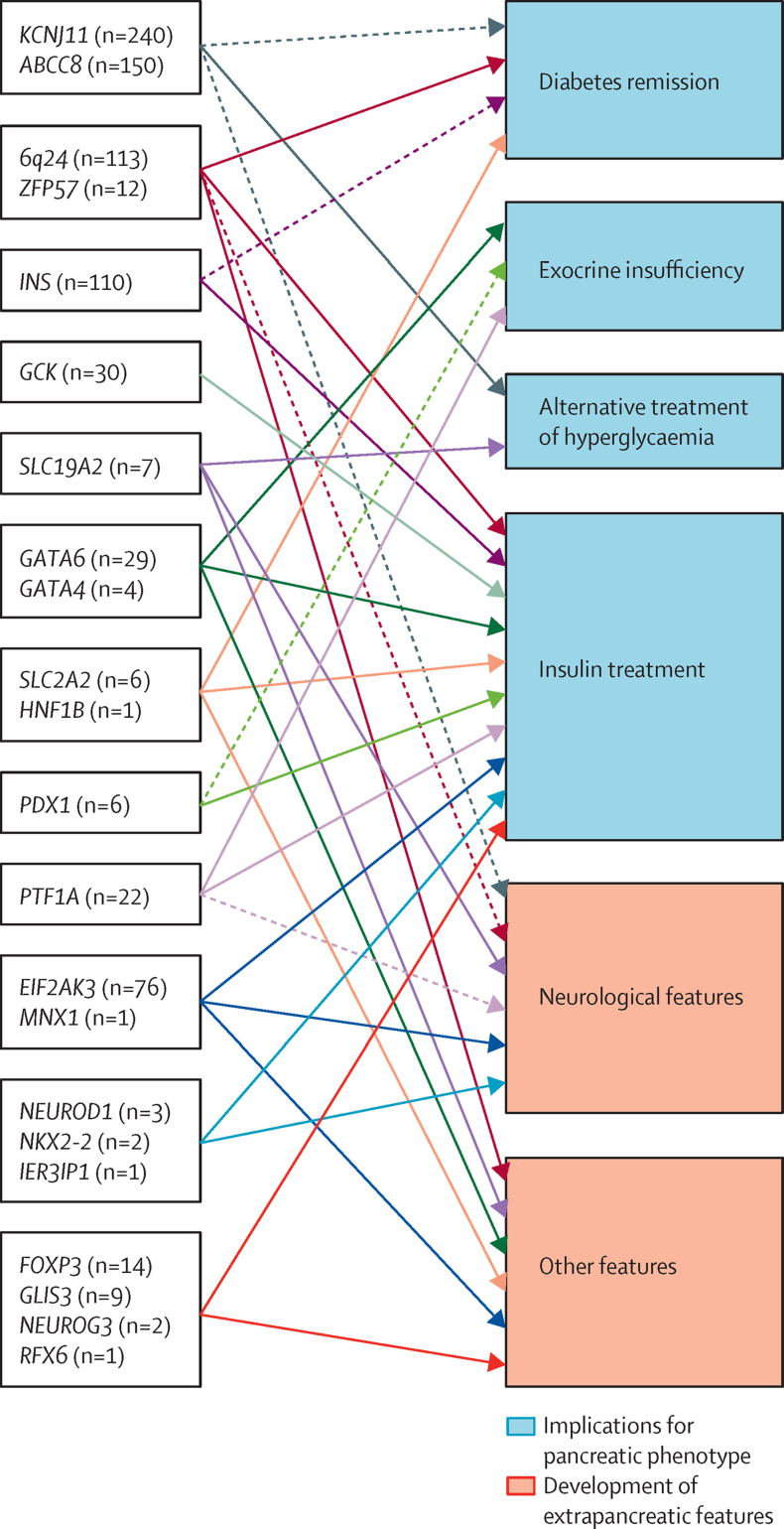
A genetic diagnosis guides clinical management Schematic representation of genetic causes of neonatal diabetes and the implications of this genetic diagnosis. N indicates the number of patients identified with mutations in each of the genes in the 1020 neonatal diabetes patient cohort. Solid arrows indicate implications for most mutations in the genes. Dashed arrows indicate the implications for specific mutations.

The different genetic causes of neonatal diabetes identified so far have a range of both pancreatic and extra-pancreatic phenotypes (figure 3). The specific subtypes have been described in detail elsewhere and are summarised in the appendix.

The pancreatic phenotypes include transient neonatal diabetes (6q24, *ABCC8, KCNJ11, INS, HNF1B, SLC2A2, ZFP57* subtypes), in which the diabetes resolves (n=219), permanent diabetes responding to sulfonylurea treatment (*KCNJ11* and *ABCC8*, n=299), permanent insulin-treated diabetes (*INS, GCK, EIF2AK3, FOXP3, GLIS3, NEUROD1, NEUROG3, NKX2-2, MNX1, IER3IP1, RFX6*, and some cases with *GATA6, GATA4* and *PDX1* mutations, n=265), and developmental disorders of the exocrine pancreas (*GATA6, PTF1A, PDX1* and *GATA4*) requiring pancreatic enzyme replacement in addition to insulin treatment (n=50). Diabetes caused by *SLC19A2* gene mutations can sometimes be successfully treated with thiamine (n=7). Therefore, the identification of the genetic cause defines the treatment requirements for the endocrine and exocrine pancreatic function.

Specific extra-pancreatic features are associated with different genetic subtypes of neonatal diabetes, with neurological features being the most common (n=184). Mutations in nine genes (*ABCC8* [22% of cases], *KCNJ11* [29% of cases], *EIF2AK3, SLC19A2, IER3IP1, PTF1A, NEUROD1, MNX1,* and *NKX2-2*) cause neonatal diabetes with neurological abnormalities. These additional features generally become evident in infancy, after diagnosis of neonatal diabetes; therefore, early genetic diagnosis in these patients predicts future development of neurological complications.

We investigated the differences in referral rate and age at referral in the referral period. The median time from the diagnosis of diabetes to referral for genetic testing showed a marked decrease from more than 4 years (240 weeks, IQR 218–4099) in 2004, to less than 3 months (10 weeks, IQR 3–23) since 2012 (appendix). The number of referrals per year was steady during the past 10 years at 80–100 per year (appendix) although there has been a shift from prevalent cases to incident cases as shown in the median duration of diabetes at referral.

**Figure 4:**
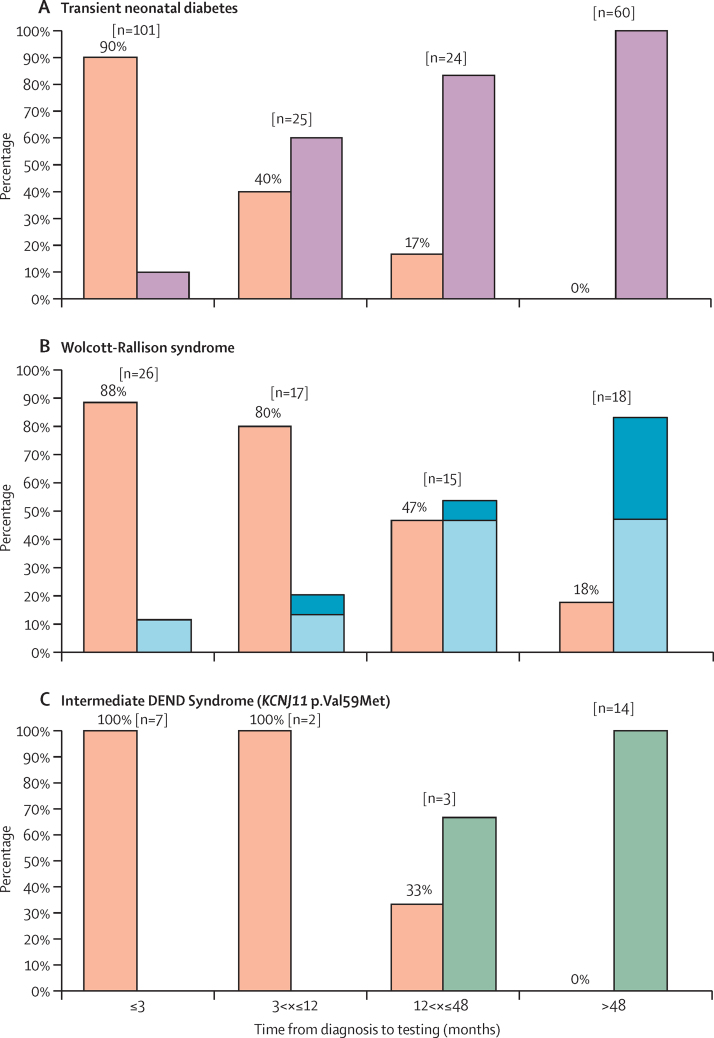
Genetic diagnosis precedes development of additional clinical features defining the neonatal diabetes subtype (A) Effect of early genetic diagnosis in transient neonatal diabetes caused by 6q24 methylation defects or potassium channel gene mutations. Bar chart representing clinical features at the time of genetic testing for neonatal diabetes. Orange=diabetes, purple=diabetes remitted. (B) The effect of age at genetic testing on whether patients have non-diabetes features of Wolcott-Rallison Syndrome at the time of referral for genetic testing. Bar chart representing clinical features at the time of genetic testing for neonatal diabetes. Orange=diabetes only, light blue=diabetes and either skeletal abnormalities or liver dysfunction, dark blue=diabetes, skeletal abnormalities, and liver dysfunction. (C) The effect of age at genetic testing on whether patients with a *KCNJ11* p.Val59Met mutation have neurological features at the time of referral for genetic testing. Bar chart representing clinical features at the time of genetic testing for neonatal diabetes. Orange=diabetes only, green=diabetes and neurological features.

We investigated the effect of decreased time from diagnosis to referral for genetic testing to see whether this resulted in patients having a genetic diagnosis before they developed typical clinical features of their genetic cause. We assessed the effect of early genetic testing in the 210 patients with a genetic diagnosis of transient neonatal diabetes caused by 6q24 methylation defects or potassium channel gene mutations (figure 4A). The likelihood that the patient's diabetes had remitted before referral was dependent on the time from diagnosis to genetic testing; only 10 (10%) of 101 patients tested early (<3 months from diagnosis) had remitted by the time of genetic testing but 60 (100%) of 60 late referrals (>48 months after diagnosis) had entered remission when genetic testing was done (p<0·0001, df=1).

We identified recessive mutations in *EIF2AK3,* which confirm a genetic diagnosis of Wolcott-Rallison syndrome, in 76 patients. Wolcott-Rallison syndrome is caused by biallelic mutations in *EIF2AK3*, a gene known to be important for regulation of endoplasmic reticulum stress.^21^ A clinical diagnosis of Wolcott-Rallison syndrome requires the presence of insulin-dependent diabetes and skeletal dysplasia or liver dysfunction.^33^ Although most of these patients are diagnosed with diabetes in the first 6 months of life, skeletal dysplasia is not evident until the infant is 1 or 2 years of age and liver dysfunction generally manifests during intercurrent illness as recurrent episodes of acute liver failure which can present at any time after the neonatal diabetes. The non-diabetes features were present only in 3 (12%) of 26 patients with early referral (<3 months from diagnosis of diabetes) but were found in most patients (15 [83%] of 18) with late referral (>48 months from diagnosis of diabetes; p<0·0001, df=1; figure 4B).

Specific mutations in *KCNJ11* and *ABCC8* cause a syndromic form of neonatal diabetes characterised by severe development delay and neurological features (DEND [Developmental delay, Epilepsy and Neonatal Diabetes] and iDEND syndrome).^5^, ^7^ The most common of these mutations is *KCNJ11* p.Val59Met which was detected in 26 patients in our cohort. Among these patients, neurological features were not present in any of those with early referral (0 of 7 referred <3 months from diagnosis) but were found in 14 (100%) of 14 patients with late referral (>48 months from diagnosis of diabetes, p<0·0001, df=1; figure 4C).

## Discussion

Patients with neonatal diabetes are referred for genetic testing close to their presentation with hyperglycaemia, often before other features have developed or are recognised. Comprehensive testing (including next-generation sequencing) of the known genetic causes of neonatal diabetes identified causal mutations in more than 80% of cases. An early genetic diagnosis predicts the best available diabetes treatment and allows anticipation of the development of related features. Therefore, neonatal diabetes is a good example of how early comprehensive genetic testing including next-generation sequencing can improve clinical management of a genetically heterogeneous disease (panel). This approach will probably have implications for many other genetically heterogeneous disorders such as hereditary hearing loss,^24^ congenital muscular dystrophy,^25^ and inherited retinal diseases.^26^ Gene panels for early genetic diagnosis of these disorders have already been developed and can be used to identify subsets of patients who are eligible for gene therapy.^26^ The paradigm shift in genetic testing has probably happened earlier for neonatal diabetes as a consequence of the availability of a free, comprehensive test and, importantly, because of the possibility of treatment change for almost 40% of patients.

Neonatal diabetes is a clinically and genetically heterogeneous disease, with 22 known genetic causes. Different genetic defects define the clinical subtypes of neonatal diabetes with important implications for clinical management and treatment (figure 3, appendix pp 9–10). About 40% of patients in our cohort have a mutation in a potassium channel subunit gene (*ABCC8* and *KCNJ11*) and most achieve improved glycaemic control upon transfer from insulin therapy to high dose sulfonylureas.^9^, ^11^

210 (20%) of 1020 patients in our cohort had a genetic diagnosis of transient neonatal diabetes caused by methylation abnormalities resulting in overexpression of the paternally inherited allele of genes at the 6q24 locus^27^, ^28^, ^29^ or mutations in *ABCC8* or *KCNJ11.*^30^ Patients with transient neonatal diabetes caused by a potassium channel mutation tend to be diagnosed and remit later than patients with 6q24 methylation abnormalities (median age at diagnosis 4 weeks in patients with potassium channel mutation *vs* 0 weeks in patients with 6q24 methylation abnormalities, median age at remission 35 weeks *vs* 13 weeks).^7^, ^31^, ^32^ With the decrease in the age at referral in our cohort, 105 (50%) of 210 transient neonatal diabetes patients were referred before diabetes remission. Of the 101 (48%) of 210 patients referred less than 3 months from diagnosis, 90% (n=91) received a genetic diagnosis of transient neonatal diabetes before diabetes remission (figure 4A). These data suggest a radical change in the role of genetic testing in neonatal diabetes, in which for most patients the genetic test predicts whether the diabetes will be transient or permanent, in addition to guiding treatment decisions (figure 3).

Neonatal diabetes is a clinical feature of 16 syndromes caused by mutations in 17 genes and in most of them it is the presenting feature. Our findings show that, during the past 10 years, the median time from clinical diagnosis to referral for genetic testing has fallen from more than 4 years to less than 3 months (appendix p 4). As a result, most patients now have genetic testing before development of the additional clinical features that characterise the syndrome. We investigated the effect of early genetic testing in the most common of these syndromes, Wolcott-Rallison syndrome. 46 (61%) of 76 patients in our cohort received a genetic diagnosis of Wolcott-Rallison syndrome before development of either skeletal dysplasia or liver failure. This proportion was even higher (89% [n=23]) for patients with early referral (<3 months from diagnosis; figure 4B). An early diagnosis of Wolcott-Rallison Syndrome is important to ensure rapid management of episodes of acute liver failure, which is a life threatening complication in these patients.

Activating mutations in *ABCC8* and *KCNJ11* are associated with a variable spectrum of phenotypes according to genotype;^34^ milder mutations cause transient neonatal diabetes, whereas mutations that severely affect the potassium channels' ability to respond to ATP concentrations cause permanent neonatal diabetes associated with neurological features (ie, intermediate DEND syndrome and DEND syndrome;^5^, ^7^, ^30^ figure 3, appendix). The most common of these mutations is the *KCNJ11* p.Val59Met, which was identified in 26 patients in our cohort. Of these patients, 100% of those referred for genetic testing less than 12 months after clinical diagnosis of diabetes had genetic testing before development of additional neurological features (figure 4C). This finding is extremely important because findings of previous studies have shown that high dose sulfonylurea therapy can improve neurological symptoms in patients with iDEND and DEND.^35^, ^36^ In these cases, an early genetic diagnosis provides clinicians with valuable information for the patients' clinical management, suggesting the possibility of treatment change and awareness of the development of neurological features.

The awareness of future development of additional clinical features as a consequence of the genetic diagnosis has important implications for patients with other neonatal diabetes-associated syndromes, such as IPEX syndrome, a severe multi-organ autoimmune disease caused by a mutation in *FOXP3.*^37^ Onset is usually within the first months of life, with one of the three cardinal features: severe enteropathy, eczema, or diabetes. An early genetic diagnosis of IPEX syndrome is crucial for clinical management and treatment because early allogenic haematopoietic stem cell transplant results in the best outcome.^38^

In conclusion, our study describes the transformation that can occur in clinical practice once genetic testing becomes the initial investigation. Traditionally, genetic testing was used to confirm a clinical diagnosis based on disease course or a cluster of clinical features. Now, early comprehensive genetic testing gives a diagnosis before the development of specific features (figure 1). The future of care in neonatal diabetes will increasingly rely on the results of genetic testing with the genetic diagnosis, not only informing a clinician of the likely course and best treatment for the diabetes, but also predicting development of additional clinical features. This model represents a new framework in which genetic testing defines, rather than just confirms, the clinical diagnosis.
