# Supplementary material for: The effect of early, comprehensive genomic testing on clinical care in neonatal diabetes: an international cohort study
Source: Lancet. 2015 Sep 5;386(9997):957–63. doi: 10.1016/S0140-6736(15)60098-8 (PMC4772451; doi:10.1016/S0140-6736(15)60098-8)
Supplement: Supplementary appendix [file mmc1.pdf]

THE LANCET

Supplementary appendix

This appendix formed part of the original submission and has been peer reviewed. We post it as supplied by the authors.

Supplement to: De Franco E, Flanagan SE, Houghton JAL, et al. The effect of early, comprehensive genomic testing on clinical care in neonatal diabetes: an international cohort study. *Lancet* 2015; published online July 29. http://dx.doi.org/10.1016/S0140-6736(15)60098-8.

Supplementary Note

Genomic testing leads clinical care in neonatal diabetes: a new paradigm

De Franco E[1], Flanagan SE[1], Houghton JAL[1], Lango Allen H[1], Mackay DJG[2,3], Temple IK[3,4], Ellard S[1] and Hattersley AT[1]

[1] Institute of Biomedical and Clinical Science, University of Exeter Medical School, EX2 5DW, UK
[2] Wessex Regional Genetics Laboratory, Salisbury Health Care Trust, Salisbury, UK
[3] Human Genetics and Genomic Medicine, Faculty of Medicine, University of Southampton, Southampton, UK
[4] Wessex Regional Genetics, Salisbury Foundation Trust and University Hospital Southampton NHS Trust, Salisbury, UK

Supplementary Figure 1. Schematic representation of the neonatal diabetes cohort. Analysis of genetic aetiologies excluded patients for whom there was insufficient DNA for comprehensive testing (n=85).

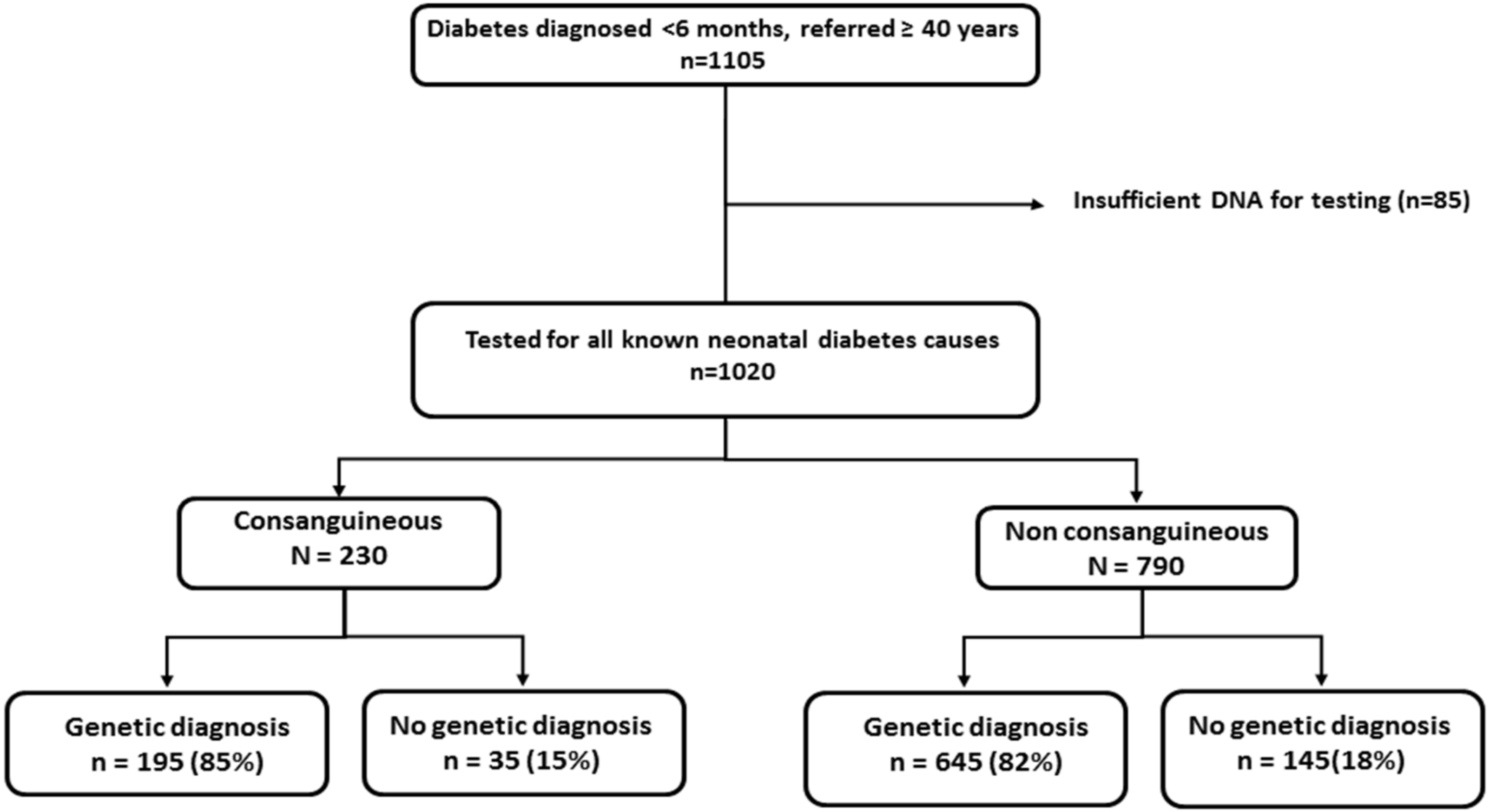

Supplementary Figure 2: Current genetic testing pipeline for neonatal diabetes referrals. Blue outline: diagnostic pipeline. Red outline: gene discovery pipeline

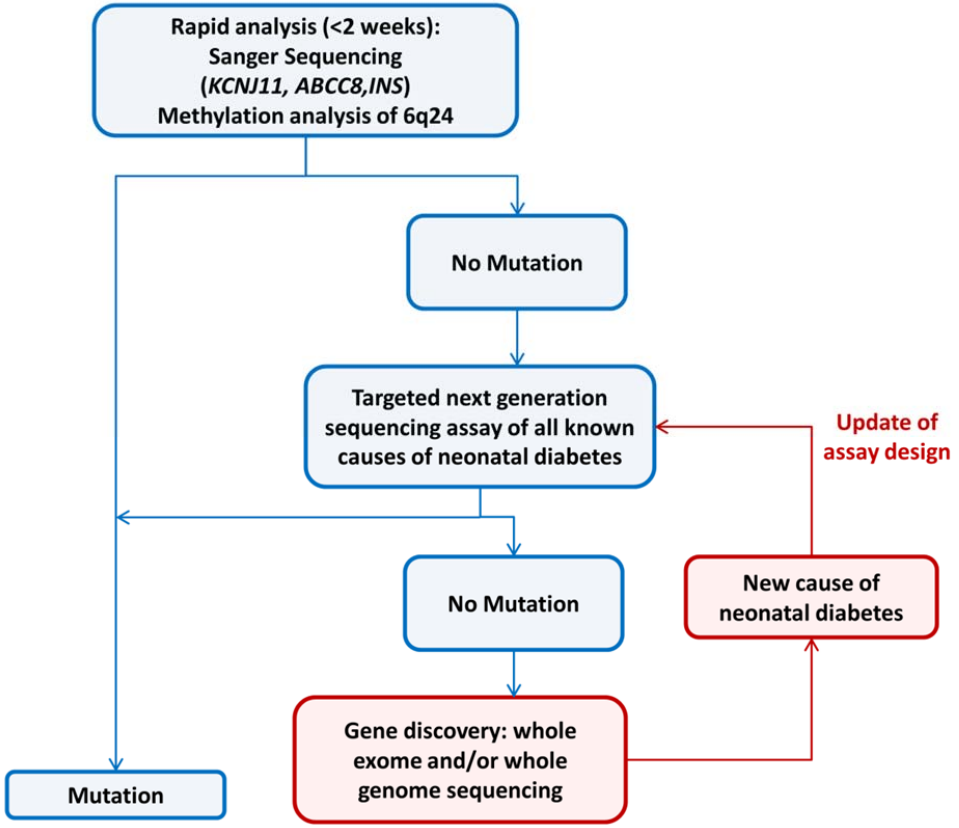

Supplementary Figure 3. Fall of the median time from diagnosis to referral for genetic testing over time. Bar chart representing the median time from clinical diagnosis of diabetes to referral for genetic testing between 01/01/2000 and 31/08/2013 (N=1016, age at diagnosis not available for N=4).

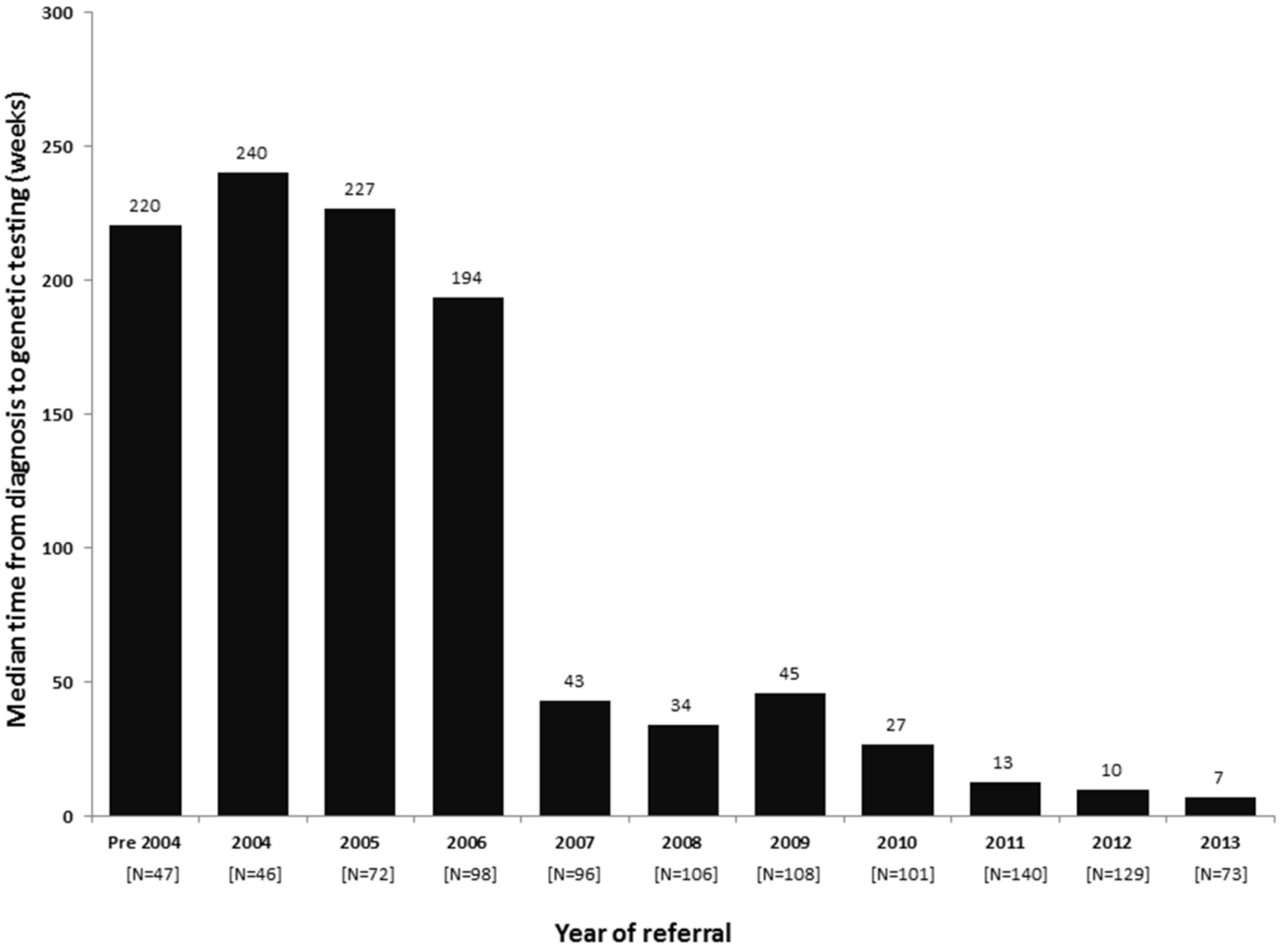

Supplementary Figure 4. Increase in the total number of referrals over time. Bar chart representing the cumulative number of worldwide referrals from 01/01/2000 to 31/08/2013 (N=1020).

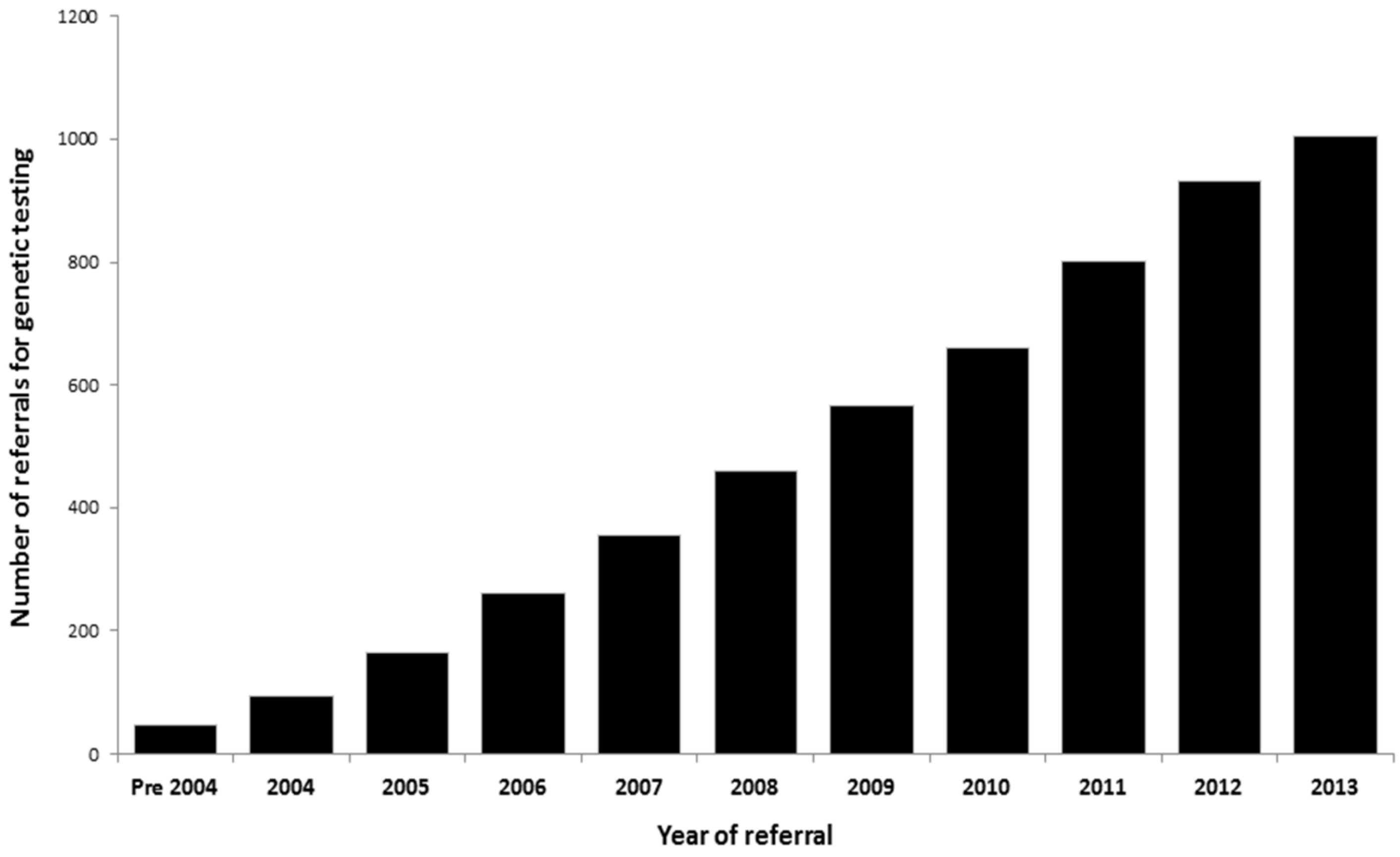

Supplementary Table 1. List of countries with high prevalence (>20%) of consanguineous unions[1] and number of referrals for neonatal diabetes testing to the Exeter Molecular Genetics laboratory

Country	Number of neonatal diabetes referrals
Bahrain	2
Bangladesh	10
Egypt	9
India	75
Iran	2
Iraq	1
Israel	6
Jordan	16
Kuwait	8
Lebanon	2
Libya	6
Morocco	8
Oman	8
Pakistan	9
Qatar	1
Saudi Arabia	36
Sudan	8
Syria	1
Tunisia	1
Turkey	83
United Arab Emirates	14

Supplementary Table 2. Number and references for 253 patients included in the cohort who have been included in previous publications by the Exeter team.

Gene	N of patients tested in Exeter and previously published	References
ABCC8	41	[2-13]
EIF2AK3	27	[3, 14-17]
FOXP3	5	[18]
GATA4	4	[19]
GATA6	25	[20-22]
GCK	28	[23]
GLIS3	4	[15, 24]
HNF1B	1	[25]
IER3IP1	0	
INS	40	[26-28]
KCNJ11	50	[3, 4, 7, 9, 29-43]
MNX1	1	[44]
NEUROD1	2	[45]
NEUROG3	1	[46]
NKX2-2	2	[44]
PDX1	3	[47]
PTF1A	11	[22, 48]
RFX6	1	[49]
SLC19A2	2	[50]
SLC2A2	5	[51]
**Total**	253	

Supplementary Table 3. Genetic causes of neonatal diabetes identified in 840 neonatal diabetes patients.

Genetic cause	Mode on inheritance	Non consanguineous N (%)	Consanguineous N (%)
**6q24**		101 (12.8%)	12 (5.2%)
ABCC8	Dominant	112 (14.2%)	3 (1.3%)
	Recessive	22 (2.8%)	13 (5.7%)
EIF2AK3	Recessive	20 (2.5%)	56 (24.3%)
FOXP3	X-linked	11 (1.4%)	3 (1.3%)
GATA4	Dominant	3 (0.4%)	1 (0.4%)
GATA6	Dominant	29 (3.7%)	0 (0.0%)
GCK	Recessive	8 (1.0%)	22 (9.6%)
GLIS3	Recessive	3 (0.4%)	6 (2.6%)
HNF1B	Dominant	2 (0.3%)	0 (0.0%)
IER3IP1	Recessive	0 (0.0%)	1 (0.4%)
INS	Dominant	77 (9.7%)	6 (2.6%)
	Recessive	9 (1.1%)	18 (7.8%)
KCNJ11	Dominant	228 (28.9%)	12 (5.2%)
MNX1	Recessive	0 (0.0%)	1 (0.4%)
NEUROD1	Recessive	1 (0.1%)	2 (0.9%)
NEUROG3	Recessive	2 (0.3%)	0 (0.0%)
NKX2-2	Recessive	0 (0.0%)	2 (0.9%)
PDX1	Recessive	2 (0.3%)	4 (1.7%)
PTF1A	Recessive	3 (0.4%)	19 (8.3%)
RFX6	Recessive	0 (0.0%)	1 (0.4%)
SLC19A2	Recessive	2 (0.3%)	5 (2.2%)
SLC2A2	Recessive	2 (0.3%)	4 (1.7%)
ZFP57	Recessive	8 (1.0%)	4 (1.7%)
**Unknown**		145 (18.4%)	35 (15.2%)
**Total**		790	230

Supplementary Table 4. Summary of the clinical features associated with the 22 neonatal diabetes subtypes. * indicates features associated to specific mutations.

Genetic cause	Neonatal Diabetes Phenotype	Diabetes Treatment	Exocrine insufficiency needing replacement therapy	Additional Features	References
**6q24**	Transient	Insulin	No	Intrauterine growth retardation, macroglossia, umbilical hernia, neurological features (rare)	52-55 56
ABCC8	Transient, Permanent	Sulfonylureas	No	Developmental delay with/without epilepsy* (22% of cases)	12, 57 56
EIF2AK3	Permanent	Insulin	No	Skeletal dysplasia, liver dysfunction, developmental delay	17, 58
FOXP3	Permanent	Insulin	No	Eczema, enteropathy, other autoimmune features	59
GATA4	Transient, Permanent	Insulin	Yes*	Congenital heart malformation	19, 60
GATA6	Transient (rare), Permanent	Insulin	Yes	Congenital heart malformation, neurological defects, hypothyroidism, gut and hepato-biliary malformation	21, 22
GCK	Permanent	Insulin	No		61-67
GLIS3	Permanent	Insulin	No	Congenital hypothyroidism, renal cysts	24, 68, 69
HNF1B	Transient	Insulin	No	Pancreatic hypoplasia, renal cysts	25, 70
IER3IP1	Permanent	Insulin	No	Microcephaly, epilepsy	71-73
INS	Transient, Permanent	Insulin	No		27, 28 56
KCNJ11	Transient, Permanent	Sulfonylureas	No	Developmental delay with/without epilepsy* (29% of cases)	35, 36 56
MNX1	Permanent	Insulin	No	Sacral agenesis, neurological defects	44
NEUROD1	Permanent	Insulin	No	Cerebellar hypoplasia, sensorineural deafness, visual impairment	45
NEUROG3	Permanent	Insulin	No	Congenital malabsorptive diarrhea	46

NKX2-2	Permanent	Insulin	No	Severe neurodevelopmental defects	[44]
PDX1	Permanent	Insulin	Yes*		[47, 74-76]
PTF1A	Permanent	Insulin	Yes	Cerebellar agenesis*	[77, 78, 48]
RFX6	Permanent	Insulin	No	Intestinal atresia and/or malrotation, gall-bladder agenesis	[49, 79]
SLC19A2	Permanent	Thiamine	No	Thiamine-responsive megaloblastic anemia, sensorineural deafness	[50, 80-82]
SLC2A2	Transient	Insulin	No	Hepato-renal glycogen accumulation, renal dysfunction, impaired utilization of glucose and galactose	[51]
ZFP57	Transient	Insulin	No	Intrauterine growth retardation, neurological features (rare)	[83, 84]

Supplementary Methods

Samples were fragmented using a Bioruptor (Diagenode, Liège, Belgium), indexed for multiplexing and hybridised (in pools of 12 samples) according to the manufacturer's instructions. Sequencing was performed with an Illumina HiSeq 2000 (Illumina, San Diego, CA, USA) (48 samples per lane) and 100 bp paired end reads. The resulting reads were aligned with BWA and duplicates were removed with Picard. We then applied GATK indel realignment, and performed SNV and INDEL discovery and genotyping using GATK UnifiedGenotyper with standard hard filtering parameters according to GATK Best Practices recommendations[84]. Variants were annotated with ANNOVAR and pathogenic mutations located within 50 bp upstream and 50 bp downstream of each exon were identified.

As previously described[85], for the 21 genes for which testing is available in the Exeter laboratory by Sanger sequencing, the average depth of coverage was over 250 reads and >99% of bases had a minimum read depth of 30. Two specific regions of low coverage (<20 reads) were observed across two ~300bp GC-rich regions in the exon 2 of *GATA6* and *GATA4*. In patients for whom these regions were not sufficiently covered and no pathogenic mutation was identified, Sanger sequencing of the specific exon 2 amplicons were carried out in patients with congenital features suggestive of a *GATA6/GATA4* mutation (e.g. low birth weight, exocrine insufficiency, congenital heart malformation). Two positive controls (for a known heterozygous deletion and a known insertion) were included in each 48 sample batch to verify the ability to detect deletions/insertions.

References for supplementary material

1. Bittles AH. A community genetics perspective on consanguineous marriage. *Community genetics* 2008; **11**(6): 324-30.
2. Abraham N, Ahamed A, Unnikrishnan AG, Kumar H, Ellard S. Permanent neonatal diabetes mellitus due to an ABCC8 mutation: a case report. *JOP : Journal of the pancreas* 2014; **15**(2): 198-200.
3. Abujbara MA, Liswi MI, El-Khateeb MS, Flanagan SE, Ellard S, Ajlouni KM. Permanent neonatal diabetes mellitus in Jordan. *Journal of pediatric endocrinology & metabolism : JPEM* 2014; **27**(9-10): 879-83.
4. Alves C, Flanagan SE, Ellard S, Mackay DJ. Clinical and molecular basis of transient neonatal diabetes mellitus in Brazilian children. *Diabetes research and clinical practice* 2012; **97**(3): e41-4.
5. Codner E, Flanagan SE, Ugarte F, et al. Sulfonylurea treatment in young children with neonatal diabetes: dealing with hyperglycemia, hypoglycemia, and sick days. *Diabetes care* 2007; **30**(5): e28-9.
6. Ellard S, Flanagan SE, Girard CA, et al. Permanent neonatal diabetes caused by dominant, recessive, or compound heterozygous SUR1 mutations with opposite functional effects. *American journal of human genetics* 2007; **81**(2): 375-82.
7. Flanagan SE, Patch AM, Mackay DJ, et al. Mutations in ATP-sensitive K+ channel genes cause transient neonatal diabetes and permanent diabetes in childhood or adulthood. *Diabetes* 2007; **56**(7): 1930-7.
8. Jain V, Flanagan SE, Ellard S. Permanent neonatal diabetes caused by a novel mutation. *Indian pediatrics* 2012; **49**(6): 486-8.
9. Klupa T, Kowalska I, Wyka K, et al. Mutations in the ABCC8 (SUR1 subunit of the K(ATP) channel) gene are associated with a variable clinical phenotype. *Clinical endocrinology* 2009; **71**(3): 358-62.
10. Oztekin O, Durmaz E, Kalay S, Flanagan SE, Ellard S, Bircan I. Successful sulfonylurea treatment of a neonate with neonatal diabetes mellitus due to a novel missense mutation, p.P1199L, in the ABCC8 gene. *Journal of perinatology : official journal of the California Perinatal Association* 2012; **32**(8): 645-7.
11. Patch AM, Flanagan SE, Boustred C, Hattersley AT, Ellard S. Mutations in the ABCC8 gene encoding the SUR1 subunit of the KATP channel cause transient neonatal diabetes, permanent neonatal diabetes or permanent diabetes diagnosed outside the neonatal period. *Diabetes, obesity & metabolism* 2007; **9 Suppl 2**: 28-39.
12. Proks P, Arnold AL, Bruining J, et al. A heterozygous activating mutation in the sulphonylurea receptor SUR1 (ABCC8) causes neonatal diabetes. *Human molecular genetics* 2006; **15**(11): 1793-800.
13. Vasanwala RF, Lim SH, Ellard S, Yap F. Neonatal Diabetes in a Singapore Children's Hospital: Molecular Diagnoses of Four Cases. *Annals of the Academy of Medicine, Singapore* 2014; **43**(6): 314-9.
14. Al-Shawi M, Al Mutair A, Ellard S, Habeb AM. Variable phenotype in five patients with Wolcott-Rallison syndrome due to the same EIF2AK3 (c.1259delA) mutation. *Journal of pediatric endocrinology & metabolism : JPEM* 2013; **26**(7-8): 757-60.
15. Habeb AM, Al-Magamsi MS, Eid IM, et al. Incidence, genetics, and clinical phenotype of permanent neonatal diabetes mellitus in northwest Saudi Arabia. *Pediatric diabetes* 2012; **13**(6): 499-505.

16. Mihci E, Turkkahraman D, Ellard S, Akcurin S, Bircan I. Wolcott-Rallison syndrome due to a novel mutation (R491X) in EIF2AK3 gene. *Journal of clinical research in pediatric endocrinology* 2012; **4**(2): 101-3.
17. Rubio-Cabezas O, Patch AM, Minton JA, et al. Wolcott-Rallison syndrome is the most common genetic cause of permanent neonatal diabetes in consanguineous families. *The Journal of clinical endocrinology and metabolism* 2009; **94**(11): 4162-70.
18. Rubio-Cabezas O, Minton JA, Caswell R, et al. Clinical heterogeneity in patients with FOXP3 mutations presenting with permanent neonatal diabetes. *Diabetes care* 2009; **32**(1): 111-6.
19. Shaw-Smith C, De Franco E, Allen HL, et al. GATA4 mutations are a cause of neonatal and childhood-onset diabetes. *Diabetes* 2014.
20. Catli G, Abaci A, Flanagan SE, et al. A novel GATA6 mutation leading to congenital heart defects and permanent neonatal diabetes: a case report. *Diabetes & metabolism* 2013; **39**(4): 370-4.
21. De Franco E, Shaw-Smith C, Flanagan SE, Shepherd MH, Hattersley AT, Ellard S. GATA6 mutations cause a broad phenotypic spectrum of diabetes from pancreatic agenesis to adult-onset diabetes without exocrine insufficiency. *Diabetes* 2013; **62**(3): 993-7.
22. Lango Allen H, Flanagan SE, Shaw-Smith C, et al. GATA6 haploinsufficiency causes pancreatic agenesis in humans. *Nature genetics* 2012; **44**(1): 20-2.
23. Raimondo A, Chakera AJ, Thomsen SK, et al. Phenotypic severity of homozygous GCK mutations causing neonatal or childhood-onset diabetes is primarily mediated through effects on protein stability. *Human molecular genetics* 2014.
24. Dimitri P, Warner JT, Minton JA, et al. Novel GLIS3 mutations demonstrate an extended multisystem phenotype. *European journal of endocrinology / European Federation of Endocrine Societies* 2011; **164**(3): 437-43.
25. Edghill EL, Bingham C, Ellard S, Hattersley AT. Mutations in hepatocyte nuclear factor-1beta and their related phenotypes. *Journal of medical genetics* 2006; **43**(1): 84-90.
26. Edghill EL, Flanagan SE, Patch AM, et al. Insulin mutation screening in 1,044 patients with diabetes: mutations in the INS gene are a common cause of neonatal diabetes but a rare cause of diabetes diagnosed in childhood or adulthood. *Diabetes* 2008; **57**(4): 1034-42.
27. Garin I, Edghill EL, Akerman I, et al. Recessive mutations in the INS gene result in neonatal diabetes through reduced insulin biosynthesis. *Proceedings of the National Academy of Sciences of the United States of America* 2010; **107**(7): 3105-10.
28. Stoy J, Edghill EL, Flanagan SE, et al. Insulin gene mutations as a cause of permanent neonatal diabetes. *Proceedings of the National Academy of Sciences of the United States of America* 2007; **104**(38): 15040-4.
29. Codner E, Flanagan S, Ellard S, Garcia H, Hattersley AT. High-dose glibenclamide can replace insulin therapy despite transitory diarrhea in early-onset diabetes caused by a novel R201L Kir6.2 mutation. *Diabetes care* 2005; **28**(3): 758-9.
30. Craig TJ, Shimomura K, Holl RW, Flanagan SE, Ellard S, Ashcroft FM. An in-frame deletion in Kir6.2 (KCNJ11) causing neonatal diabetes reveals a site of interaction between Kir6.2 and SUR1. *The Journal of clinical endocrinology and metabolism* 2009; **94**(7): 2551-7.
31. Dupont J, Pereira C, Medeira A, Duarte R, Ellard S, Sampaio L. Permanent neonatal diabetes mellitus due to KCNJ11 mutation in a Portuguese family: transition from insulin to oral sulfonylureas. *Journal of pediatric endocrinology & metabolism : JPEM* 2012; **25**(3-4): 367-70.
32. Edghill EL, Gloyn AL, Gillespie KM, et al. Activating mutations in the KCNJ11 gene encoding the ATP-sensitive K+ channel subunit Kir6.2 are rare in clinically defined type 1 diabetes diagnosed before 2 years. *Diabetes* 2004; **53**(11): 2998-3001.

33. Flanagan SE, Edghill EL, Gloyn AL, Ellard S, Hattersley AT. Mutations in KCNJ11, which encodes Kir6.2, are a common cause of diabetes diagnosed in the first 6 months of life, with the phenotype determined by genotype. *Diabetologia* 2006; **49**(6): 1190-7.
34. Gloyn AL, Cummings EA, Edghill EL, et al. Permanent neonatal diabetes due to paternal germline mosaicism for an activating mutation of the KCNJ11 Gene encoding the Kir6.2 subunit of the beta-cell potassium adenosine triphosphate channel. *The Journal of clinical endocrinology and metabolism* 2004; **89**(8): 3932-5.
35. Gloyn AL, Pearson ER, Antcliff JF, et al. Activating mutations in the gene encoding the ATP-sensitive potassium-channel subunit Kir6.2 and permanent neonatal diabetes. *The New England journal of medicine* 2004; **350**(18): 1838-49.
36. Gloyn AL, Reimann F, Girard C, et al. Relapsing diabetes can result from moderately activating mutations in KCNJ11. *Human molecular genetics* 2005; **14**(7): 925-34.
37. Ioannou YS, Ellard S, Hattersley A, Skordis N. KCNJ11 activating mutations cause both transient and permanent neonatal diabetes mellitus in Cypriot patients. *Pediatric diabetes* 2011; **12**(2): 133-7.
38. Khadilkar VV, Khadilkar AV, Kapoor RR, Hussain K, Hattersley AT, Ellard S. KCNJ11 activating mutation in an Indian family with remitting and relapsing diabetes. *Indian journal of pediatrics* 2010; **77**(5): 551-4.
39. Letha S, Mammen D, Valamparampil JJ. Permanent neonatal diabetes due to KCNJ11 gene mutation. *Indian journal of pediatrics* 2007; **74**(10): 947-9.
40. Shimomura K, Horster F, de Wet H, et al. A novel mutation causing DEND syndrome: a treatable channelopathy of pancreas and brain. *Neurology* 2007; **69**(13): 1342-9.
41. Siklar Z, Ellard S, Okulu E, et al. Transient neonatal diabetes with two novel mutations in the KCNJ11 gene and response to sulfonylurea treatment in a preterm infant. *Journal of pediatric endocrinology & metabolism : JPEM* 2011; **24**(11-12): 1077-80.
42. Slingerland AS, Hurkx W, Noordam K, et al. Sulphonylurea therapy improves cognition in a patient with the V59M KCNJ11 mutation. *Diabetic medicine : a journal of the British Diabetic Association* 2008; **25**(3): 277-81.
43. Tammaro P, Flanagan SE, Zadek B, et al. A Kir6.2 mutation causing severe functional effects in vitro produces neonatal diabetes without the expected neurological complications. *Diabetologia* 2008; **51**(5): 802-10.
44. Flanagan SE, De Franco E, Lango Allen H, et al. Analysis of transcription factors key for mouse pancreatic development establishes NKX2-2 and MNX1 mutations as causes of neonatal diabetes in man. *Cell metabolism* 2014; **19**(1): 146-54.
45. Rubio-Cabezas O, Minton JA, Kantor I, Williams D, Ellard S, Hattersley AT. Homozygous mutations in NEUROD1 are responsible for a novel syndrome of permanent neonatal diabetes and neurological abnormalities. *Diabetes* 2010; **59**(9): 2326-31.
46. Rubio-Cabezas O, Jensen JN, Hodgson MI, et al. Permanent Neonatal Diabetes and Enteric Anendocrinosis Associated With Biallelic Mutations in NEUROG3. *Diabetes* 2011; **60**(4): 1349-53.
47. De Franco E, Shaw-Smith C, Flanagan SE, et al. Biallelic PDX1 (insulin promoter factor 1) mutations causing neonatal diabetes without exocrine pancreatic insufficiency. *Diabetic medicine : a journal of the British Diabetic Association* 2013; **30**(5): e197-200.
48. Weedon MN, Cebola I, Patch AM, et al. Recessive mutations in a distal PTF1A enhancer cause isolated pancreatic agenesis. *Nature genetics* 2014; **46**(1): 61-4.

49. Smith SB, Qu HQ, Taleb N, et al. Rfx6 directs islet formation and insulin production in mice and humans. *Nature* 2010; **463**(7282): 775-80.
50. Shaw-Smith C, Flanagan SE, Patch AM, et al. Recessive SLC19A2 mutations are a cause of neonatal diabetes mellitus in thiamine-responsive megaloblastic anaemia. *Pediatric diabetes* 2012; **13**(4): 314-21.
51. Sansbury FH, Flanagan SE, Houghton JA, et al. SLC2A2 mutations can cause neonatal diabetes, suggesting GLUT2 may have a role in human insulin secretion. *Diabetologia* 2012; **55**(9): 2381-5.
52. Gardner RJ, Mackay DJ, Mungall AJ, et al. An imprinted locus associated with transient neonatal diabetes mellitus. *Human molecular genetics* 2000; **9**(4): 589-96.
53. Temple IK, Gardner RJ, Robinson DO, et al. Further evidence for an imprinted gene for neonatal diabetes localised to chromosome 6q22-q23. *Human molecular genetics* 1996; **5**(8): 1117-21.
54. Temple IK, Shield JP. Transient neonatal diabetes, a disorder of imprinting. *Journal of medical genetics* 2002; **39**(12): 872-5.
55. Thomas IH, Saini NK, Adhikari A, et al. Neonatal diabetes mellitus with pancreatic agenesis in an infant with homozygous IPF-1 Pro63fsX60 mutation. *Pediatric diabetes* 2009; **10**(7): 492-6.
56. Busiah K, Drunat S, Vaivre-Douret L, et al. Neuropsychological dysfunction and developmental defects associated with genetic changes in infants with neonatal diabetes mellitus: a prospective cohort study [corrected]. *The lancet Diabetes & endocrinology* 2013; **1**(3): 199-207.
57. Babenko AP, Polak M, Cave H, et al. Activating mutations in the ABCC8 gene in neonatal diabetes mellitus. *The New England journal of medicine* 2006; **355**(5): 456-66.
58. Delepine M, Nicolino M, Barrett T, Golamaully M, Lathrop GM, Julier C. EIF2AK3, encoding translation initiation factor 2-alpha kinase 3, is mutated in patients with Wolcott-Rallison syndrome. *Nature genetics* 2000; **25**(4): 406-9.
59. Chatila TA, Blaeser F, Ho N, et al. JM2, encoding a fork head-related protein, is mutated in X-linked autoimmunity-allergic disregulation syndrome. *The Journal of clinical investigation* 2000; **106**(12): R75-81.
60. D'Amato E, Giacopelli F, Giannattasio A, et al. Genetic investigation in an Italian child with an unusual association of atrial septal defect, attributable to a new familial GATA4 gene mutation, and neonatal diabetes due to pancreatic agenesis. *Diabetic medicine : a journal of the British Diabetic Association* 2010; **27**(10): 1195-200.
61. Njolstad PR, Sovik O, Cuesta-Munoz A, et al. Neonatal diabetes mellitus due to complete glucokinase deficiency. *The New England journal of medicine* 2001; **344**(21): 1588-92.
62. Barbetti F, Cobo-Vuilleumier N, Dionisi-Vici C, et al. Opposite clinical phenotypes of glucokinase disease: Description of a novel activating mutation and contiguous inactivating mutations in human glucokinase (GCK) gene. *Molecular endocrinology (Baltimore, Md)* 2009; **23**(12): 1983-9.
63. Bennett K, James C, Mutair A, Al-Shaikh H, Sinani A, Hussain K. Four novel cases of permanent neonatal diabetes mellitus caused by homozygous mutations in the glucokinase gene. *Pediatric diabetes* 2011; **12**(3 Pt 1): 192-6.
64. Durmaz E, Flanagan S, Berdeli A, et al. Variability in the age at diagnosis of diabetes in two unrelated patients with a homozygous glucokinase gene mutation. *Journal of pediatric endocrinology & metabolism : JPEM* 2012; **25**(7-8): 805-8.
65. Osbak KK, Colclough K, Saint-Martin C, et al. Update on mutations in glucokinase (GCK), which cause maturity-onset diabetes of the young, permanent neonatal diabetes, and hyperinsulinemic hypoglycemia. *Human mutation* 2009; **30**(11): 1512-26.

66. Rubio-Cabezas O, Diaz Gonzalez F, Aragones A, Argente J, Campos-Barros A. Permanent neonatal diabetes caused by a homozygous nonsense mutation in the glucokinase gene. *Pediatric diabetes* 2008; **9**(3 Pt 1): 245-9.
67. Turkkahraman D, Bircan I, Tribble ND, Akcurin S, Ellard S, Gloyn AL. Permanent neonatal diabetes mellitus caused by a novel homozygous (T168A) glucokinase (GCK) mutation: initial response to oral sulphonylurea therapy. *The Journal of pediatrics* 2008; **153**(1): 122-6.
68. Senee V, Chelala C, Duchatelet S, et al. Mutations in GLIS3 are responsible for a rare syndrome with neonatal diabetes mellitus and congenital hypothyroidism. *Nature genetics* 2006; **38**(6): 682-7.
69. Taha D, Barbar M, Kanaan H, Williamson Balfe J. Neonatal diabetes mellitus, congenital hypothyroidism, hepatic fibrosis, polycystic kidneys, and congenital glaucoma: a new autosomal recessive syndrome? *American journal of medical genetics Part A* 2003; **122a**(3): 269-73.
70. Yorifuji T, Kurokawa K, Mamada M, et al. Neonatal diabetes mellitus and neonatal polycystic, dysplastic kidneys: Phenotypically discordant recurrence of a mutation in the hepatocyte nuclear factor-1beta gene due to germline mosaicism. *The Journal of clinical endocrinology and metabolism* 2004; **89**(6): 2905-8.
71. Poulton CJ, Schot R, Kia SK, et al. Microcephaly with simplified gyration, epilepsy, and infantile diabetes linked to inappropriate apoptosis of neural progenitors. *American journal of human genetics* 2011; **89**(2): 265-76.
72. Abdel-Salam GM, Schaffer AE, Zaki MS, et al. A homozygous IER3IP1 mutation causes microcephaly with simplified gyral pattern, epilepsy, and permanent neonatal diabetes syndrome (MEDS). *American journal of medical genetics Part A* 2012; **158a**(11): 2788-96.
73. Shalev SA, Tenenbaum-Rakover Y, Horovitz Y, et al. Microcephaly, epilepsy, and neonatal diabetes due to compound heterozygous mutations in IER3IP1: insights into the natural history of a rare disorder. *Pediatric diabetes* 2014; **15**(3): 252-6.
74. Nicolino M, Claiborn KC, Senee V, Boland A, Stoffers DA, Julier C. A novel hypomorphic PDX1 mutation responsible for permanent neonatal diabetes with subclinical exocrine deficiency. *Diabetes* 2010; **59**(3): 733-40.
75. Schwitzgebel VM, Mamin A, Brun T, et al. Agenesis of human pancreas due to decreased half-life of insulin promoter factor 1. *The Journal of clinical endocrinology and metabolism* 2003; **88**(9): 4398-406.
76. Stoffers DA, Zinkin NT, Stanojevic V, Clarke WL, Habener JF. Pancreatic agenesis attributable to a single nucleotide deletion in the human IPF1 gene coding sequence. *Nature genetics* 1997; **15**(1): 106-10.
77. Al-Shammari M, Al-Husain M, Al-Kharfy T, Alkuraya FS. A novel PTF1A mutation in a patient with severe pancreatic and cerebellar involvement. *Clinical genetics* 2011; **80**(2): 196-8.
78. Sellick GS, Barker KT, Stolte-Dijkstra I, et al. Mutations in PTF1A cause pancreatic and cerebellar agenesis. *Nature genetics* 2004; **36**(12): 1301-5.
79. Spiegel R, Dobbie A, Hartman C, de Vries L, Ellard S, Shalev SA. Clinical characterization of a newly described neonatal diabetes syndrome caused by RFX6 mutations. *American journal of medical genetics Part A* 2011; **155a**(11): 2821-5.
80. Bay A, Keskin M, Hizli S, Uygun H, Dai A, Gumruk F. Thiamine-responsive megaloblastic anemia syndrome. *International journal of hematology* 2010; **92**(3): 524-6.
81. Bergmann AK, Sahai I, Falcone JF, et al. Thiamine-responsive megaloblastic anemia: identification of novel compound heterozygotes and mutation update. *The Journal of pediatrics* 2009; **155**(6): 888-92.e1.

82. Mandel H, Berant M, Hazani A, Naveh Y. Thiamine-dependent beriberi in the "thiamine-responsive anemia syndrome". *The New England journal of medicine* 1984; **311**(13): 836-8.
83. Mackay DJ, Callaway JL, Marks SM, et al. Hypomethylation of multiple imprinted loci in individuals with transient neonatal diabetes is associated with mutations in ZFP57. *Nature genetics* 2008; **40**(8): 949-51.
84. Mackay DJ, Temple IK. Transient neonatal diabetes mellitus type 1. *American journal of medical genetics Part C, Seminars in medical genetics* 2010; **154c**(3): 335-42.
